# Zipper plot: visualizing transcriptional activity of genomic regions

**DOI:** 10.1186/s12859-017-1651-7

**Published:** 2017-05-02

**Authors:** Francisco Avila Cobos, Jasper Anckaert, Pieter-Jan Volders, Celine Everaert, Dries Rombaut, Jo Vandesompele, Katleen De Preter, Pieter Mestdagh

**Affiliations:** 1 Center for Medical Genetics, Ghent University, De Pintelaan 185, Ghent, Belgium; 2 Cancer Research Institute Ghent, De Pintelaan 185, Ghent, Belgium; 3 Bioinformatics Institute Ghent from Nucleotides to Networks, De Pintelaan 185, Ghent, Belgium

## Abstract

**Background:**

Reconstructing transcript models from RNA-sequencing (RNA-seq) data and establishing these as independent transcriptional units can be a challenging task. Current state-of-the-art tools for long non-coding RNA (lncRNA) annotation are mainly based on evolutionary constraints, which may result in false negatives due to the overall limited conservation of lncRNAs.

**Results:**

To tackle this problem we have developed the Zipper plot, a novel visualization and analysis method that enables users to simultaneously interrogate thousands of human putative transcription start sites (TSSs) in relation to various features that are indicative for transcriptional activity. These include publicly available CAGE-sequencing, ChIP-sequencing and DNase-sequencing datasets. Our method only requires three tab-separated fields (chromosome, genomic coordinate of the TSS and strand) as input and generates a report that includes a detailed summary table, a Zipper plot and several statistics derived from this plot.

**Conclusion:**

Using the Zipper plot, we found evidence of transcription for a set of well-characterized lncRNAs and observed that fewer mono-exonic lncRNAs have CAGE peaks overlapping with their TSSs compared to multi-exonic lncRNAs. Using publicly available RNA-seq data, we found more than one hundred cases where junction reads connected protein-coding gene exons with a downstream mono-exonic lncRNA, revealing the need for a careful evaluation of lncRNA 5′-boundaries. Our method is implemented using the statistical programming language R and is freely available as a webtool.

**Electronic supplementary material:**

The online version of this article (doi:10.1186/s12859-017-1651-7) contains supplementary material, which is available to authorized users.

## Background

The introduction of RNA-sequencing (RNA-seq) has revolutionized the field of molecular biology, revealing that up to 75% of the human genome is actively transcribed [1]. The majority of this transcriptome consists of so-called long non-coding RNAs (lncRNAs). Reconstructing accurate transcript models for these lncRNAs is a major challenge when processing RNA-seq data. In general, lncRNA transcripts are less abundant compared to protein coding genes [2], often resulting in a lack of junction reads from which transcript models are inferred. In addition, lncRNAs are frequently located in the vicinity of protein coding genes and could therefore represent unannotated extensions of untranslated regions (UTRs) rather than independent transcriptional units. Finally, transcript reconstruction from RNA-seq data often gives rise to large numbers of single-exon transcripts. Distinguishing single-exon fragments that represent independent transcriptional units from those that result from genomic DNA contamination or incomplete transcript assembly is not straightforward.

State-of-the-art tools for lncRNA annotation based on evolutionary constraints such as PLAR (pipeline for lncRNA annotation from RNA-seq data) [3] and slncky [4], might filter out some putative lncRNA transcripts depending on stringent conservation criteria. PLAR removes transcripts that are short (< 2 kb) and lowly expressed (FPKM < 5) and focuses on the annotation of syntenic lncRNAs. Given the limited conservation of lncRNAs [5] and given that both tools exclude any transcript that partially or totally overlaps protein-coding genes, such approaches may result in a large number of false negatives.

LncRNA transcript models can be refined and filtered by integrating complementary datasets on chromatin state (i.e. ChIP sequencing (ChIP-seq) for histone marks or DNase sequencing (DNase-seq)) and transcript boundaries (i.e. CAGE sequencing (CAGE-seq) to mark the transcription start site (TSS) or 3P-seq to mark the 3′ end of poly-adenylated transcripts) [6]. Transcripts for which the transcription start site coincides with a CAGE-peak and is in close proximity to a H3K4me3 or H3K27ac mark are more likely to be independent transcriptional units compared to transcripts that lack these features.

GRIT [7] is a command line-based tool that uses CAGE in conjunction with RNA-seq data but does not take advantage of other important layers of genomic information such as open chromatin (DNase-seq) and histone marks (ChIP-seq data) typically associated with active transcription.

To tackle the challenge of establishing lncRNAs as independent transcriptional units we have created the Zipper plot, a novel visualization and analysis method available as a quick and user-friendly webtool [8] that employs publicly available CAGE-seq, ChIP-seq and DNase-seq data across a large collection of tissue and cell types. The user only needs to provide a list of genomic features (one per line), each consisting of three tab-separated fields: chromosome, human genomic coordinate (hg19) of the TSS and strand. Our webtool will retrieve the closest CAGE-seq/DNase-seq/ChIP-seq peak to each TSS for thousands of genomic features at the same time. The closer these peaks are, the higher the evidence of independent transcriptional activity for the set of genomic features.

## Results and discussion

### Implementing the Zipper plot as a webtool

The Zipper plot is freely available as a webtool (front-end) at [8] and has been implemented using the JavaScript library jQuery, PHP and HTML5. The back-end (server) contains a peak-based database (see Methods) and the necessary code to retrieve and sort the closest CAGE-seq/ChIP-seq/DNase-seq peak to each TSS, to create the plot (see “Zipper plot construction”) and to compute several statistics to assess the TSS-peak associations (see “Summary statistics and generation of html summary reports”). This code was written using the R statistical programming language [9] along with the data.table [10], ggplot2 [11], knitr [12], R.utils [13], grid [9] and gridExtra [14] packages. The communication between the web interface and our server is established using PHP.

Due to memory constraints on our server, we limited the number of genomic features per input file to 20,000. However, to allow users to integrate our tool as part of bigger pipelines, we have made our scripts available at Github [15].

### Database querying

To start using the webtool, the user only needs to upload a list of genomic features (one per line), each consisting of three tab-separated fields: chromosome, human genomic coordinate (hg19) of the TSS and strand. Optionally, users can provide an additional fourth column containing labels for the genomic features being studied.

If the user has a file from another genomic build (e.g. hg38), we propose two alternatives to convert it to hg19: 1) hgLiftOver [16]: a webtool where users can upload a file with “chrN:start-end” or BED format and select the new genomic build of interest; 2) CrossMap [17]: a tool that supports more file types as input, including BAM, SAM and BigWig among others. Detailed information about its usage and download can be found at [18].

Importantly, hgLiftOver can also be installed locally on unix-based systems by downloading the executable [19] and appropriate chain files [20].

In a second step, the user has to select the data type of interest among the ones available in our database (CAGE-seq, ChIP-seq or DNase-seq peaks; see Methods) and has the option to run the analysis in one sample type of interest or across all available sample types. In the first option, the user knows in advance in which tissue the set of genomic features are more likely to be expressed; with the second option, each individual genomic feature is analyzed across all samples and the sample in which the peak is most closely associated to the genomic feature is retained for further analysis. Importantly, all CAGE-peaks are used by default but the user can set a more stringent threshold if desired (tags per million mapped reads (tpm) > 0). A detailed user guide can be found at [21].

### Zipper plot construction

Once the user’s input is uploaded to our website and the data type of interest has been selected, the data.table package [10] is used to sort TSSs from the user’s input in a chromosome-wise manner and to perform a fast binary search (O(log n) time) in compiled C to retrieve the closest ChIP-seq/DNase-seq/CAGE-seq peak to each TSS. It retrieves the “start” and “end” genomic coordinates of the closest peak, always considering the “start” as the part of the peak closest to the TSS. The Additional file 1: Methods (“Definition of the distance between a TSS and the closest peak” section) contain three different examples on how these coordinates are determined.

**Fig. 1:**
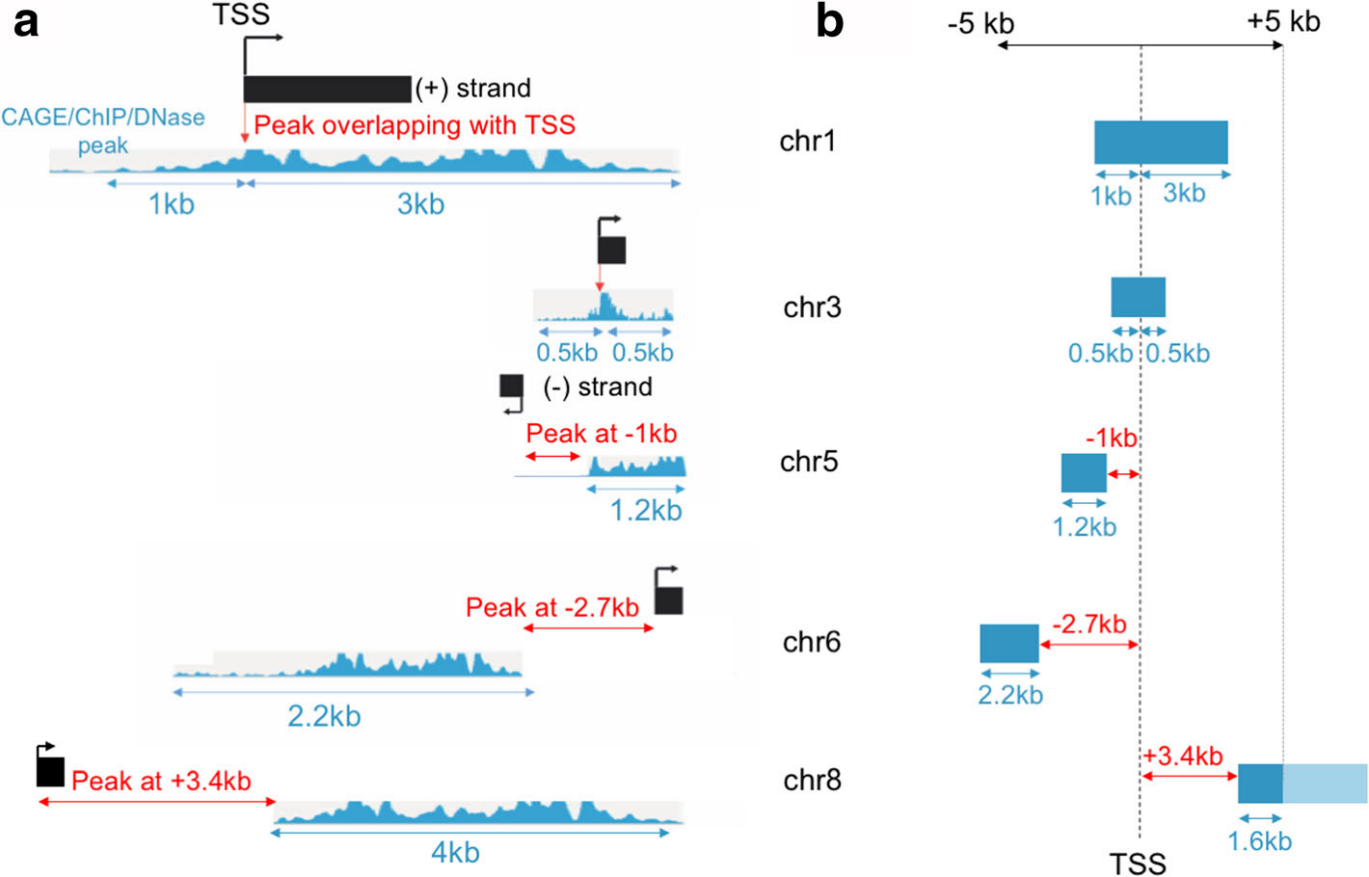
The closest CAGE-seq/ChIP-seq/DNase-seq peak to each TSS is rapidly retrieved using a binary search. **a** The process of finding the closest CAGE peak takes into account the strand information supplied by the user (ChIP-seq and DNase-seq data are unstranded). If a TSS is located on the positive DNA strand (TSSs on chromosomes 1, 3, 6 and 8), peaks with a genomic coordinate greater than the TSS are considered downstream (=positive distance) of the genomic feature. If a TSS is located on the negative DNA strand (third TSS on chromosome 5), peaks with a genomic coordinate greater than the TSS are considered upstream (=negative distance) of the genomic feature. Peak widths and overall peak enrichment for each region (signalValue for ChIP-seq and DNase-seq data; tpm expression values for CAGE-seq) are simultaneously retrieved. **b** Once the distances to the closest peaks have been retrieved they are ordered and placed on top of a vertical axis representing the TSS. Since the Zipper plot is visualized (by default) in a 5 kb window, peaks that are wider than 5 kb or are further away from the TSS will not appear (i.e. TSS on chromosome 8; darker region will appear whereas the faded region exists but it is not displayed)

The peaks are then ranked based on the distance from the TSS to the “start” of the closest peak and a Zipper plot is generated with the aid of the ggplot2 package [11]: peaks overlapping with the TSS are placed at the top of the plot and the zipper starts to open as the peaks are located further away from the TSSs. By default, the Zipper plot is visualized in a +/− 5 kilobase (kb) window around the TSS but the window size can be adjusted by the user. Figure 1 shows in detail how the Zipper plot is built.

### Summary statistics and generation of html summary reports

In parallel with the construction of every Zipper plot, two statistics named Zipper Height (ZH) and Area Under the Zipper (AUZ) are calculated. ZH corresponds to the quotient between the number of genomic features with a peak overlapping with the TSS and the total number of genomic features being studied (ZH ∈ [0,1]). The AUZ_global is computed as the sum of all the areas between the closest peak and the TSS of each genomic feature (for a detailed explanation﻿ ﻿see “Definition of the sum of all areas between the closest peak and the TSS” and “Small AUZ values, areas in the plot and how AUZ_window is calculated (Fig. 3d)” in the Additional file 1: Methods).

However, since the distribution of peaks upstream or downstream of the TSSs can be asymmetric, AUZleft (sum of all the areas for cases where the closest peak was found upstream the TSS) and AUZright (sum of all the areas for cases where the closest peak was found downstream the TSS) are considered independently (see “Rationale for calculating both positive and negative distances between closest peaks and TSSs” in Additional file 1: Methods for more details).

The closer the peaks are distributed around the TSSs, the smaller the AUZ and the higher the evidence of independent transcriptional activity for the set of genomic features. A “closed zipper” (AUZ = 0) indicates an overlap between the closest peak and TSS for all the genomic features being studied. We have also incorporated the AUZ_window, which depends on the window size choice (by default +/− 5 kb) and is computed using only those peaks that lie within the window. The method virtually sets to 5 kb (or other value if the user changes the default window size) all those distances that are located more than 5 kb away from the TSS. This allows a quick visual comparison between two Zipper plots built using the same window size. Following the same reasoning as the paragraph above, we have incorporated both AUZ_window_right and AUZ_window_left separately. Of note, ZH and AUZ are negatively correlated.

A one-sided *p*-value (AUZ_pval) is calculated by comparing the AUZ of the Zipper plot built with the user’s input to 100 (by default) or 1000 random Zipper plots created by selecting as many random locations as the number of genomic features supplied by the user while maintaining the same distribution of TSSs per chromosome. Since truly random locations picked uniformly along the length of each chromosome are not representative of possible lncRNA TSSs, we have excluded from the selection those genomic regions containing gaps, centromeres, telomeres, heterochromatin and repetitive regions from [22, 23] using the BEDTools suite [24]. The *p*-value is computed dividing the number of random cases with AUZ values smaller than or equal to the AUZ for the user case by the total number of repetitions. The *p*-value represents the chance of finding a random Zipper plot with an AUZ_global smaller than or equal to the AUZ_global of the actual use case or, in other words, whether it is likely that the set of TSSs chosen by the user was randomly selected or not. Therefore, the smaller the *p*-value, the higher the likelihood your set of genomic features are truly independent transcriptional units.

When evaluating genomic features in one sample type, the closest peaks in that sample type are retrieved for both the random TSSs and the user input. Optionally, the closest peak in each sample type can be retrieved for each TSS and, for each TSS, a TSS *p*-value is calculated comparing how many tissues have a peak as close (or closer) to the TSS than the one found in the tissue chosen by the user.

On the other hand, if the user selects all sample types, the closest peaks among all possible sample types are retrieved for both the random TSS and the user input. AUZs are calculated and a *p*-value is calculated similarly to the case where the user selects one sample type.

Eventually, the knitr package [12] is used to generate an html report containing 1) the Zipper plot; 2) all the aforementioned parameters/statistics; 3) a summary table listing closest peaks, peak widths and overall peak enrichment information.

### Validation and applications of the Zipper plot

**Fig. 2:**
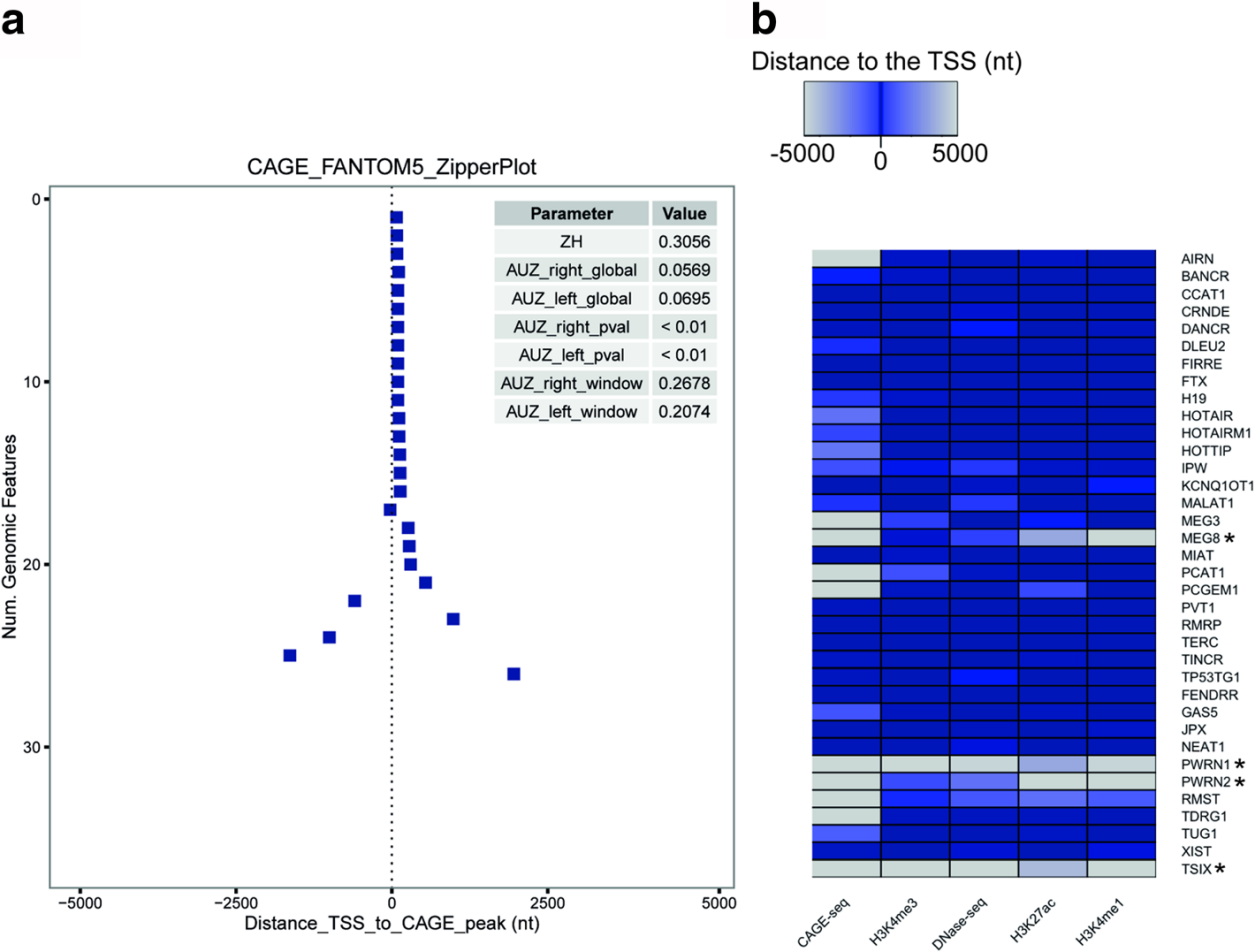
There is evidence of transcriptional activity for 32 out of 36 well-characterized lncRNAs using the Zipper plot. **a** Zipper plot and associated statistics for the set of 36 well-characterized lncRNAs proposed by [4] using CAGE-seq data. Even though the visualization contains a +/− 5 kb window, it is clear that the closest CAGE peaks for 26 lncRNAs are within +/− 2.5 kb from the TSS. Both AUZ_right_pval and AUZ_*left*_pval are smaller than 0.01, suggesting that the set of TSSs are more closely associated with CAGE peaks compared to random regions in the genome. **b** Heatmap showing the distance between TSSs and CAGE-seq, DNase-seq, H3K4me1, H3K4me3 and H3K27ac peaks. *Darker colours* represent peaks that are closer to the TSSs. LncRNAs marked with an *asterisk* do not have enough evidence of transcriptional activity. (nt = nucleotides)

To assess the usefulness of our webtool, we first investigated a set of 36 well-characterized lncRNAs proposed by [4]. The Zipper plot created using only the FANTOM5 (CAGE-seq) data showed that 26 out of 36 lncRNAs have a CAGE peak within +/− 5 kb from their TSSs in at least one of the sample types present in our database (Fig. 2a; detailed output available in Additional file 2: Table S1). Moreover, when also including H3K4me3 and DNaseI (marks for active transcription and open chromatin) together with H3K4me1 and H3K27ac (marks for active enhancer RNAs), 32 out of 36 lncRNAs have peaks within +/-﻿ 5 kb from their TSSs (Fig. 2b). These results demonstrate that, while most of the well-characterized lncRNAs have evidence for transcription initiation at or near their presumed TSS, some may be incompletely annotated with respect to their TSS. This is especially apparent from the CAGE-seq Zipper plot (Fig. 2a).

**Fig. 3:**
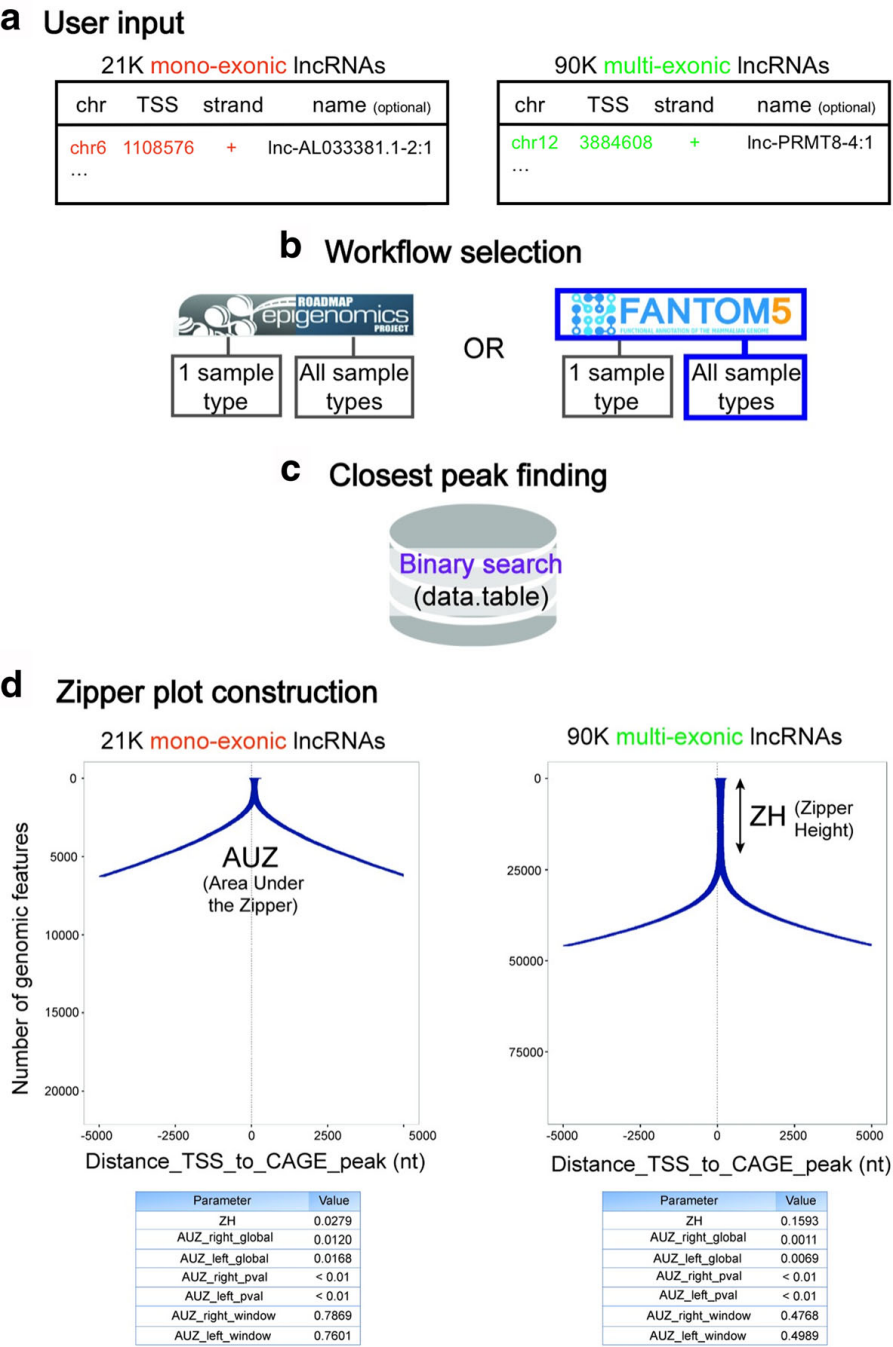
Fewer mono-exonic lncRNAs have CAGE-seq peaks overlapping with their TSSs compared to multi-exonic lncRNAs. This is reflected in smaller Zipper Height (ZH) and higher Area Under the Zipper (AUZ) values. **a**) As described in the “Database querying” section, users may provide an additional fourth column in the input file with labels for each TSS (optional). **b**) FANTOM5 data (CAGE-seq) and “All sample types” workflow was selected. **c**) ﻿The data.table package was﻿ used to retrieve the closest ﻿CAGE-seq peak to each TSS.﻿ **d**) Peaks are ranked based on the distance from the TSS to the closest peak and a Zipper plot is generated. Since both plots are visualized in a +/− 5 kb window, AUZ_window values can be directly compared: smaller values (multi-exonic lncRNAs) represent higher evidence of independent transcriptional activity for the set of genomic features being studied. This conclusion can also be made looking at the ZH values: a bigger ZH value means a higher proportion of lncRNAs with a CAGE peak overlapping with the TSS. Finally, both AUZ_*right*_pval and AUZ_*left*_pval are smaller than 0.01, so it is unlikely that the set of TSSs from mono and multi-exonic lncRNAs were randomly selected

As a second example application of the Zipper plot, we evaluated the transcriptional independence of all human lncRNAs listed in Lncipedia 3.1 [25]. We studied the distribution of the closest CAGE-seq peaks (FANTOM5 data) around the TSSs of all mono-exonic and all multi-exonic human lncRNA transcripts (21,102 and 90,508 respectively) (Fig. 3a–c) and found that 589 mono-exonic lncRNAs (2.8%) presented a CAGE-peak overlapping with the TSS and 6256 (29.7%) had a peak within a +/− 5 kb window. On the other hand, 14,419 multi-exonic lncRNAs (15.9%) presented a CAGE-peak overlapping with the TSS and 45,878 (50.7%) had a peak within a +/− 5 kb window (Fig. 3d). These differences, also reflected in greater AUZ_global values in the former case, suggest that numerous mono-exonic lncRNAs might not be truly independent transcriptional units.

We hypothesized that at least a fraction of mono-exonic lncRNAs were actually extensions of UTRs from upstream protein coding genes or genomic DNA contamination. To further investigate this hypothesis, we first retrieved the intron lengths for all RefSeq protein coding genes (hg19; using the UCSC Table Browser data retrieval tool) [26, 27] and found that 80% of them are smaller than or equal to 5827 nucleotides. In a second step, we artificially “stitched” mono-exonic lncRNAs that do not have a CAGE peak within 500 nucleotides from their TSSs to the 3′ end of any protein coding gene located within 5827 nucleotides on the same strand. This process led to 536 mono-exonic lncRNAs stitched to upstream protein coding genes.

If these lncRNAs were truly unannotated portions of upstream coding genes, we should find junction reads spanning one exon from a protein coding gene and another exon from a lncRNA. To evaluate this, we used RNA-seq data from The Cancer Genome Atlas (TCGA) [28, 29] and Universal Human Reference RNA (UHRR) samples [30, 31] (see Methods). Since junction reads that are shared between exons of overlapping lncRNAs and protein coding genes cannot be assigned unambiguously, they were excluded from the analyses. Next, we established a minimum of at least one junction read linking a lncRNA to an upstream protein coding gene and a minimum overlap of two nucleotides between the junction read and the protein coding gene exon and a minimum overlap of two nucleotides between the junction read and the lncRNA exon.

Strikingly, we found spanning reads for 135 out of the 536 cases (25.19%) based on the TCGA RNA-seq data and for 35 (6.53%) based on UHRR RNA-seq data (Additional file 3: Table S2).

We also tried to stitch multi-exonic lncRNAs that do not have a CAGE peak within 500 nucleotides from their TSSs in the same manner as we did for mono-exonic lncRNAs, resulting in 675 multi-exonic lncRNAs stitched to upstream protein coding genes. We found spanning reads for 127 out of the 675 cases (18.81%) based on the TCGA RNA-seq data and for 33 (4.89%) based on UHRR RNA-seq data (Additional file 3: Table S2). Of all the junction reads from the TCGA RNA-seq data fou﻿nd to span a protein coding gene and a lncRNA, 92.59% of them﻿ entirely overlap with protein coding gene exons and 88.15% of them entirely overlap with lncRNA exons. On the other hand, 89.31% of the junction reads from UHRR RNA-seq data entirely overlap with a protein coding gene exons and 91.91% of the junction reads entirely overlap with lncRNA exons.

Both TCGA and UHRR samples shared junction reads for 34 mono-exonic and 31 multi-exonic lncRNAs stitched to an upstream protein coding gene. Table 1 shows the distribution of junction reads spanning a protein coding gene and downstream lncRNA based on the TCGA RNA-seq data.

**Table 1:** Distribution of junction reads (JR) from 1460 TCGA samples connecting protein-coding gene exons with a downstream mono and multi-exonic lncRNA

	1 < = JR < = 10	11 < = JR < = 100	JR > 100	Total
Protein coding gene + mono-exonic lncRNA	86	37	12	135
Protein coding gene + multi-exonic lncRNA	81	31	15	127

These junction reads suggest that the latter are actually extensions of untranslated regions from upstream protein coding genes. Detailed information for each individual case can be found on Additional file 3: Table S2

These results support our hypothesis and reveal the need for a careful evaluation of lncRNA 5′-boundaries using CAGE-seq data and histone marks as demonstrated here or alternative procedures such as 5′-RACE(-seq) [32].

To further expand the applicability of our tool, we plan to integrate publicly available data from methods that detect nascent RNAs (GRO-seq and PRO-seq), to extend the number of samples when new data becomes available and to allow users to work with their own data.

## Conclusion

We have created the Zipper plot, a novel visualization and analysis method available as a webtool [8] that allows researchers to quickly evaluate the reliability of the annotation of thousands of novel transcripts and lncRNAs at the same time. Using the Zipper plot we found evidence of transcription for a set of well-characterized lncRNAs and observed that fewer mono-exonic lncRNAs have CAGE peaks overlapping with their TSSs compared to multi-exonic lncRNAs. Using publicly available RNA-seq data, we discovered more than one hundred cases where junction reads connected protein-coding gene exons with a downstream mono-exonic lncRNA, revealing the need for a careful evaluation of lncRNA boundaries.

We also recognize a limitation in our webtool: the presence of a CAGE-peak and activating histone marks at the TSS is indicative of independent transcription, but the absence of such features does not imply the opposite. Low abundant transcripts may not show up in the CAGE-seq data because of too low sequencing depth or the expression of the lncRNA may be restricted to a tissue of cell type not (yet) included in the CAGE-seq, ChIP-seq and DNase-seq database. Importantly, TSSs of RNA transcripts reconstructed from RNA-seq data might appear several nucleotides downstream of a CAGE-seq peak. Particularly for low abundant RNA transcripts, this inconsistency may be the result of an incomplete transcript assembly due to non-uniformity of read coverage towards 5′ ends and should be carefully examined.

## Methods

### Establishing a peak-based database using publicly available datasets

ChIP-seq & DNase-seq from 127 consolidated human epigenomes already processed in the context of the Roadmap Epigenomics Project (111 from NIH Roadmap Epigenomics Mapping Consortium (Release 9 of the Human Epigenome Atlas) [33] and 16 cell line epigenomes from the ENCODE Project Consortium [34, 35]) were retrieved from the “Peak Calling” section at [36]. DNase-seq and ChIP-seq data consists of ENCODE narrowPeak, broadPeak and gappedPeak files. Detailed information about these formats can be found at [37].

These files contain lists of peaks that were obtained by a peak caller algorithm in the context of the Roadmap Epigenomics Project. The peak calling process identified regions in the genome that were enriched with aligned reads (“peaks”) as a consequence of the ChIP or DNase-seq experiment.

We focused our filtering approach on the qValue, being a measurement of statistical significance for the signal enrichment of each peak using the false discovery rate (FDR). We set a FDR < = 0.05, implying that only those peaks with qValue < = 0.05 were retained in our database for downstream applications.

The following activating marks [38] were used to construct the database: marks for open chromatin (DNaseI); acetylation marks commonly found in actively transcribed promoters (H3K27ac, H3K9ac, and H3K14ac), methylation marks found in actively transcribed promoters (H3K4me1, H3K4me2, H3K4me3 and H4K20me1) and modifications added as consequence of transcription (H3K36me3, H3K79me2 at 5′ end of gene bodies) adding up to more than 134 million peaks. (Additional file 4: Table S3).

CAGE-seq expression data (RLE normalized) for human samples was retrieved from the Functional Annotation of the Mammalian Genome (FANTOM5) project [39, 40]. CAGE-seq measures expression by means of sequencing from the 5′ end (transcription start site (TSS)) of capped molecules. In case of multiple replicates per sample type, only one replicate was retained, bringing the total number of samples to 649 with a total of 200,737 peaks. (Additional file 5: Table S4).

### Obtaining junction reads from publicly available RNA-seq data

One thousand four hundred sixty RNA-seq samples from TCGA across different cancer types [28, 29] (See Additional file 6: Table S5 for detailed information on cancer type and TCGA barcodes) and 80 UHRR samples from the Sequencing Quality Control (SEQC) project publicly available at the Gene Expression Omnibus (GEO) database with accession number GSE47774 (Sample A: Replicates 1–4; Beijing Genomics Institute) [30, 31] were mapped to the human genome (GRCh37) using TopHat2 [41] with default parameters, resulting in 279,507,060 and 12,679,075 junction reads respectively.

## Additional files

Additional file 1: Methods. (PDF 1792 kb)

Additional file 2: Table S1. Summary table for the set of 36 well-characterized lncRNAs using CAGE-seq data. (XLS 69 kb)

Additional file 3: Table S2. Junction reads between protein coding genes and mono/multi-exonic lncRNAs based on RNA-seq data from TCGA and UHRR; nucleotides of junction read overlapping with lncRNA and protein coding gene exons. (XLS 65 kb)

Additional file 4: Table S3. Correspondence between Roadmap Epigenomics names and actual sample types; number of peaks and number of epigenomes available for each case; peak width and peak enrichment distributions across chromosomes for narrow, broad and gapped peaks (for each mark). (XLS 109 kb)

Additional file 5: Table S4. Correspondence between FANTOM5 names and actual sample types; number of CAGE-seq peaks per chromosome; peak width and tpm distributions across chromosomes. (XLS 101 kb)

Additional file 6: Table S5. Cancer type and barcode for each sample from TCGA. (XLS 138 kb)

Additional file 7: Table S6. HGNC, Ensembl ID, PMID, chromosome location, TSS and strand information for the set of 36 well-characterized lncRNAs. (XLS 26 kb)

## Abbreviations

3P-seq: Poly(A)-Position Profiling by Sequencing
AUZ: Area Under the Zipper
CAGE-seq: Cap Analysis of Gene Expression sequencing
ChIP-seq: Chromatin Immunoprecipitation sequencing
DNase-seq: DNase sequencing
FANTOM5: Functional Annotation of Mammalian Genomes 5
FDR: False Discovery Rate
FPKM: Fragments Per Kilobase Million
GRO-seq: Global Run-On Sequencing
*H3K14ac*: *Histone H3 lysine 14 acetylation*
*H3K27ac*: *Histone H3 lysine 27 acetylation*
*H3K36me3*: *Histone H3 lysine 36 trimethylation*
*H3K4me1*: *Histone H3 lysine 4 monomethylation*
*H3K4me2*: *Histone H3 lysine 4 dimethylation*
*H3K4me3*: *Histone H3 lysine 4 trimethylation*
*H3K79me2*: *Histone H3 lysine 79 dimethylation*
*H3K9ac*: *Histone H3 lysine 9 acetylation*
*H4K20me1*: *Histone H4 lysine 20 monomethylation*
lncRNA: Long non-coding RNA
PRO-seq: Precision nuclear Run-On sequencing
RACE-seq: Rapid amplification of cDNA ends sequencing
RNA-seq: RNA sequencing
SEQC: Sequencing Quality Control
TCGA: The Cancer Genome Atlas
tpm: tags per million mapped reads
TSS: Transcription Start Site
UHRR: Universal Human Reference RNA
ZH: Zipper Height
