# Supplementary material for: Zipper plot: visualizing transcriptional activity of genomic regions
Source: BMC Bioinformatics. 2017 May 2;18:231. doi: 10.1186/s12859-017-1651-7 (PMC5414305; doi:10.1186/s12859-017-1651-7)
Supplement: Supplementary file 1 — (PDF 1792 kb) [file 12859_2017_1651_MOESM1_ESM.pdf]

Additional file 1: Methods

Definition of the distance between a TSS and the closest peak

Each peak consists of two genomic coordinates delimiting its start and end positions. Regardless of whether there is a peak overlapping with the TSS or not, the data.table package retrieves the “start” and “end” genomic coordinates of the closest ChIP-seq/DNase-seq/CAGE-seq peak as depicted in this figure, **with the “start” always being the part of the peak closest to the TSS**:

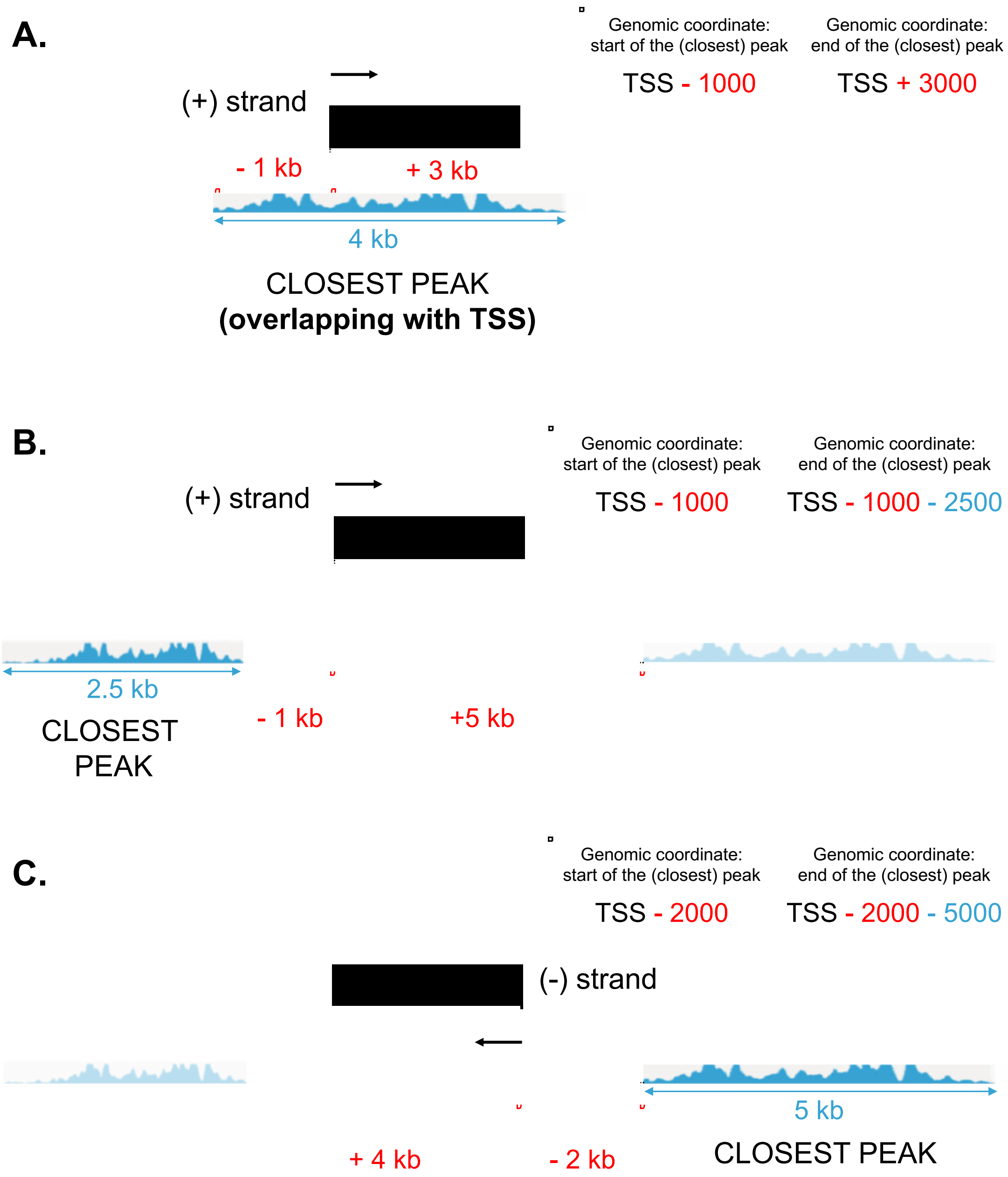

“A” represents a case where the closest peak overlaps with the TSS whereas “B” and “C” are cases where it does not. In “B”, the closest peak is the one on the left hand side, since a distance of 1kb is smaller than 5kb. In “C”, the closest peak is the one on the right hand side, since a distance of 2kb is smaller than 4kb.

Finally, **peaks are ranked based on the distance from the TSS to the “start” of the closest peak** and a Zipper plot is generated as described in the main manuscript.

Rationale for calculating both positive and negative distances between closest peaks and TSSs

Thurman and colleagues [1] demonstrated that, for 56 different cell types, a common pattern for the distribution of the H3K4me3 and DNase marks around the TSS can been observed: DNase marks were located a few nucleotides upstream the TSS whereas the H3K4me3 appeared several nucleotides downstream the TSS (Fig. 3a-b from [1*]).

We have investigated this hypothesis as an argument to support the need of calculating AUZleft (negative distances between TSSs and closest peak) and AUZright (positive distances between TSSs and closest peak).

We used all lncRNAs (mono and multi-exonic) from Lncipedia 3.1 and retrieved 11,989 lncRNAs (unique TSSs) with a CAGE peak overlapping with the TSS. Next, we retrieved the closest H3K4me3 and DNase peaks (narrowPeak; across all sample types) for those and generated the following Zipper plots:

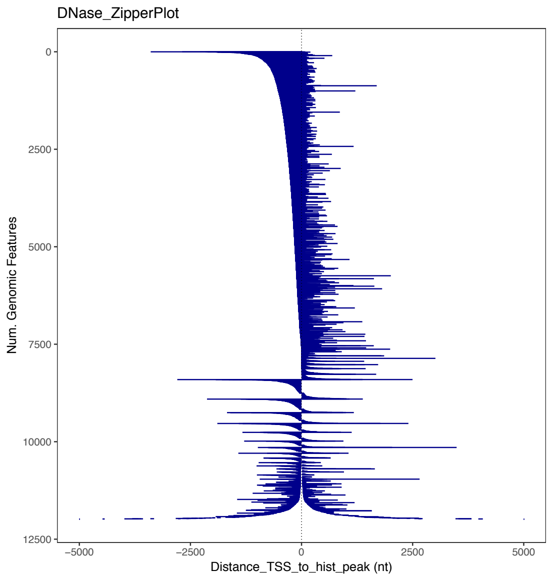

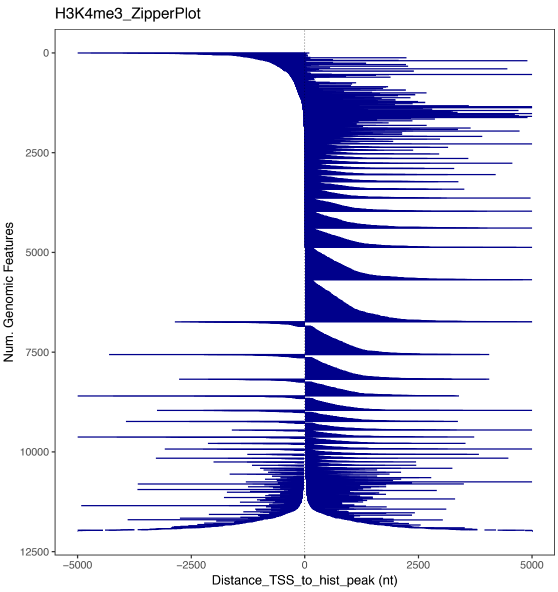

From these plots is clear that the DNase marks (left) were mostly found a few nucleotides upstream the TSS (=negative distances in the plot; OX axis) whereas the H3K4me3 (right) were mostly found several nucleotides downstream the TSS (=positive distances in the plot; OX axis), in agreement with what was observed in [1*].

Finally, we plotted the distribution of distances between these two marks and found that for 9,177 lncRNAs (76.54%) the H3K4me3 mark was found downstream the DNase mark (=distances greater than 0):

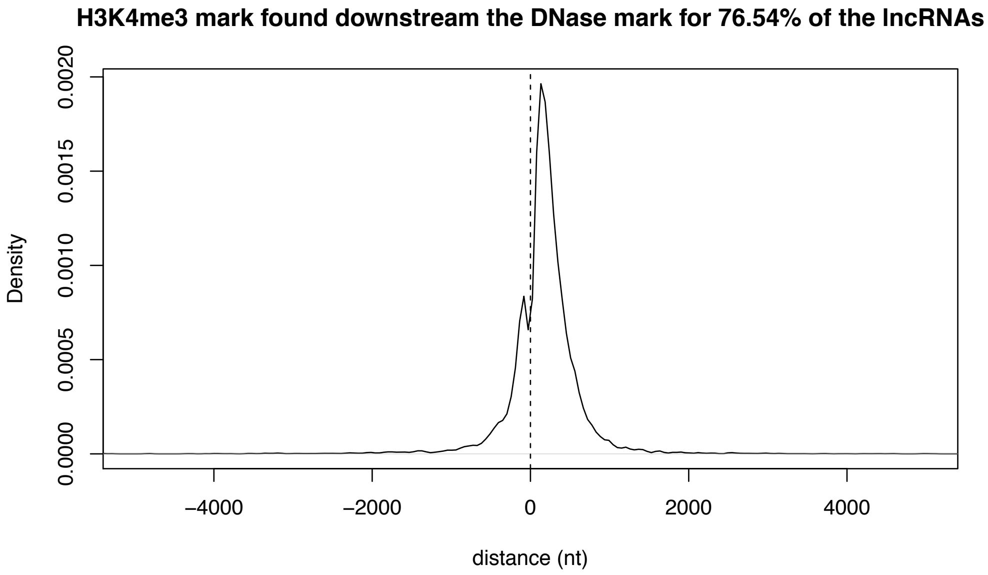

This result is a useful example where differences between positive and negative distances are clear.

Definition of the sum of all areas between the closest peak and the TSS

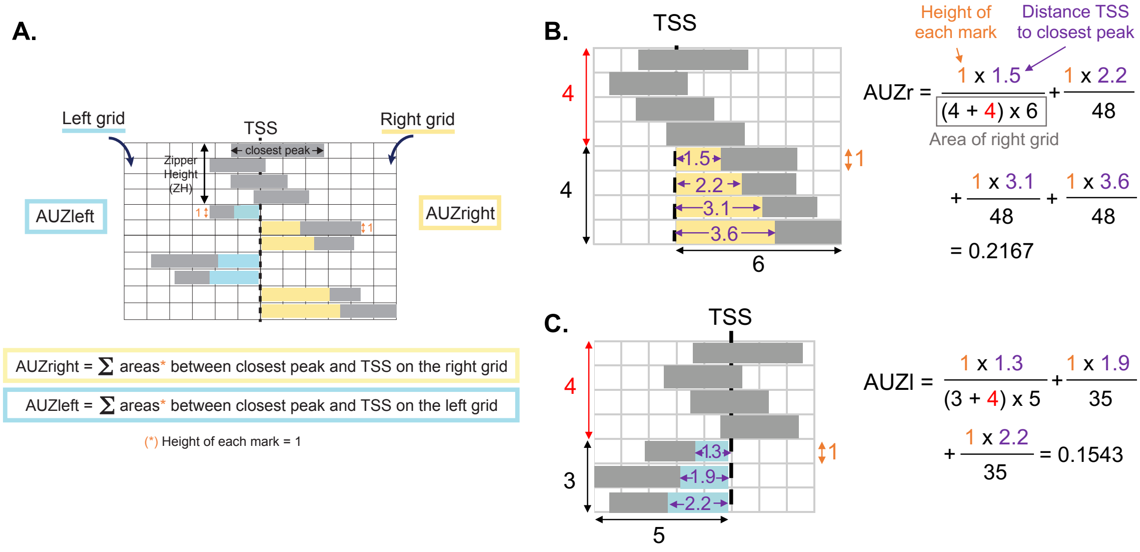

A) AUZ is computed as the sum of all the areas between the closest peak and the TSS of each genomic feature. Since the distribution of peaks upstream or downstream of the TSSs can be asymmetric, AUZleft and AUZright are considered independently. B) shows how the AUZ_right is computed; C) shows how AUZ_left is computed.

Importantly, the width of the grid for each Zipper plot is determined by the (closest) peak furthest away from the TSS among all retrieved ones. Looking at the image below, AUZ_{CASE_1} seems bigger than AUZ_{CASE_2} while it should be smaller (marks in CASE_1 are closer to TSS than marks in CASE_2):

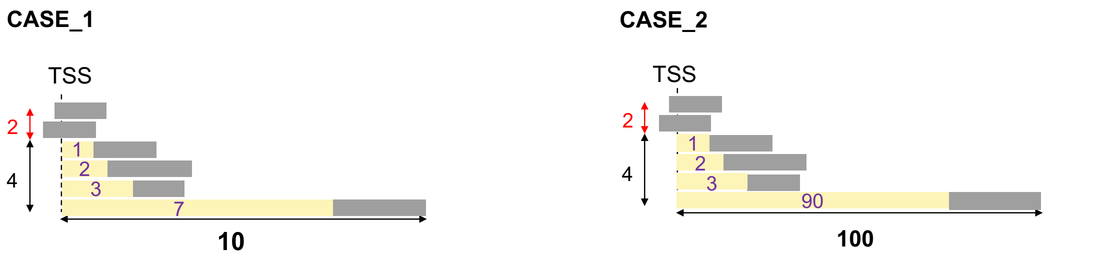

$$AUZ_{CASE\_1} = \frac{1\times1}{(2+4)\times10} + \frac{2\times1}{60} + \frac{3\times1}{60} + \frac{7\times1}{60} = 0.2167$$

$$AUZ_{CASE\_2} = \frac{1\times1}{(2+4)\times100} + \frac{2\times1}{600} + \frac{3\times1}{600} + \frac{90\times1}{600} = 0.16$$

This issue is due to the difference in grid areas: to compare two AUZ values directly, both need to come from a grid with the same width. This can be achieved by re-scaling the AUZ from the case (or cases, if we are comparing more than 2 Zipper plots) with the

smallest width by the ratio between both grids (and maintaining the AUZ from the case with biggest width unchanged):

$AUZ_{CASE\_1\_rescaled} = AUZ_{CASE\_1\_original} \times (Grid_{CASE\_1} / Grid_{CASE\_2}) = 0.2167 * (60/600) = 0.02167$

The resulting AUZ values are: $AUZ_{CASE\_1\_rescaled} = 0.02167 < AUZ_{CASE\_2\_unchanged} = 0.16$, as expected.

This re-scaling formula is used during the computation of the AUZ values of Zipper plots constructed from random regions. More specifically, to compute the AUZ_pval.

Small AUZ values, areas in the plot and how AUZ_window is calculated (Fig 3D)

As explained in the previous section, the width of the grid for each Zipper plot is determined by the (closest) peak furthest away from the TSS among all retrieved ones. Regarding the Zipper plot for the 21,000 mono-exonic lncRNAs, we found that there are few cases where the closest CAGE-seq peak is several Mb away (x-axis) from the TSS. If we also take into account that we are studying thousands of lncRNAs simultaneously (y-axis), this results in a grid area of the order of 10^9. Since 76.24% of the mono-exonic lncRNAs have a CAGE peak within 50kb from the TSS (very small value compared to 1Mb), this resulted in very small AUZ_global values.

The "actual areas in the plot" correspond to the AUZ_window values. By default, the Zipper plot is visualized in a +/- 5kb window. In the case of the plot for the 21,000 mono-exonic lncRNAs, the method virtually sets to 5kb (or other value if the user changes the default window size) all those distances that are located more than 5kb away from the TSS. Therefore, the y-axis extends down to 21,000.

AUZ_window values correspond to the AUZ value that users can "visually" infer from the plot visualized in the pre-defined (5kb) window.

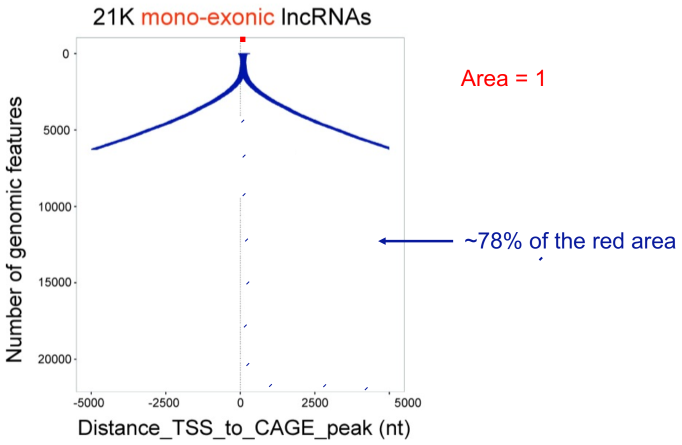

Parameter	Value
ZH	0.0279
AUZ_right_global	0.0120
AUZ_left_global	0.0168
AUZ_right_pval	< 0.01
AUZ_left_pval	< 0.01
AUZ_right_window	0.7869
AUZ_left_window	0.7601

References

1*. Thurman RE, Rynes E, Humbert R, et al. The accessible chromatin landscape of the human genome. Nature. 2012;489(7414):75-82. doi:10.1038/nature11232.
